# A quantitative and qualitative analysis of patient group narratives suggests common biopsychosocial red flags of undiagnosed rare disease

**DOI:** 10.1186/s13023-024-03143-8

**Published:** 2024-04-19

**Authors:** Mariam Al-Attar, Sondra Butterworth, Lucy McKay

**Affiliations:** 1 Salford Royal Hospital, Northern Care Alliance NHS Foundation Trust, Salford, UK; 2 RareQol Ltd, Unit 1 Edison Court, Ellice Way, Wrexham, LL13 7YT UK; 3 Medics4RareDiseases, Unit 12 Treadaway Technical Centre, Treadaway Hill, High Wycombe Loudwater, HP10 9RS UK

**Keywords:** Rare disease, Biopsychosocial, Diagnostic delay, Diagnostic odyssey, Clinical presentation, Red flags, Patient survey, Patient experience, Narrative medicine, Mixed methods research

## Abstract

**Background:**

The ‘diagnostic odyssey’ is a common challenge faced by patients living with rare diseases and poses a significant burden for patients, their families and carers, and the healthcare system. The diagnosis of rare diseases in clinical settings is challenging, with patients typically experiencing a multitude of unnecessary tests and procedures. To improve diagnosis of rare disease, clinicians require evidence-based guidance on when their patient may be presenting with a rare disease. This study aims to identify common experiences amongst patients with rare diseases, to inform a series of ‘red flags’ that can aid diagnosis of rare diseases in non-specialist settings.

A questionnaire was developed by Medics for Rare Diseases, informed by the experiences of clinicians, rare disease patients and patient advocates, and was shared with UK-based rare disease patient groups. Study participants were engaged via social media platforms, blogs and email newsletters of three umbrella rare disease organisations. The questionnaire, comprising 22 questions, was designed to identify typical experiences relating to physical and psychosocial manifestations and presentation of disease, patient interactions with healthcare providers, and family history.

**Results:**

Questionnaire responses were received from 79 different rare disease patient groups and the common experiences identified were used to inform seven red flags of rare disease: multi-system involvement (3 or more); genetic inheritance pattern; continued presentation throughout childhood and adulthood; difficulties at school, especially relating to absences, difficulty participating in physical education and experiences of bullying or social isolation; multiple specialist referrals; extended period with unexplained symptoms; and misdiagnosis. In light of the red flags identified, recommendations for primary care and education settings have been proposed, focusing on the need for holistic assessment and awareness of both physical and psychosocial factors.

**Conclusions:**

This study identified key commonalities experienced by patients with rare disease across physical and psychosocial domains, in addition to understanding patients’ history and experiences with healthcare providers. These findings could be used to develop a clinical decision‑making tool to support non-specialist practitioners to consider when their patient may have an undiagnosed rare condition, which may minimise the challenges of the ‘diagnostic odyssey’ and improve the patient experience.

**Supplementary Information:**

The online version contains supplementary material available at 10.1186/s13023-024-03143-8.

## Background

Rare diseases are a heterogenous group of conditions defined by the European Union Orphan Drugs Regulation as those affecting fewer than one individual per 2,000 within the general population [1]. Whilst each individual disease is rare, there are an estimated 6,000 rare conditions globally [2], making rare diseases as a whole common in any population. For example, the global prevalence of rare diseases is 3.5–5.9% which, in the UK, amounts to over 3.5 million people [2, 3]. Around 80% of rare diseases have a genomic component to their aetiology, with causative genetic variants documented in around 4,000 different genes to date [4].

It is typical for patients with rare disease to experience an extended ‘diagnostic odyssey’ between the appearance of first symptoms and receipt of a diagnosis [5]. During this period patients often experience unnecessary tests and procedures, and delays in receiving appropriate care, which can have negative impacts on quality of life [5, 6]. Furthermore, patients frequently receive incorrect diagnoses and inappropriate treatments which can result in medical, psychological and social and economic impacts, such as the progression of symptoms and loss of confidence in medicine [1]. The ‘diagnostic odyssey’ can also have a significant impact on the emotional and mental wellbeing of patients’ families [5]; the uncertainty of the diagnosis period can be overwhelming and stressful, especially when there is limited information available regarding the expected prognosis of the disease [5]. Undiagnosed rare diseases also pose a significant cost to the UK National Health Service; costs associated with patients with undiagnosed rare disease have amounted to over £3.4 billion within the last 10 years [7]. Patients with rare disease have also been found to visit hospitals more frequently than the general population, incurring greater resource and treatment costs that overall contribute to an average individual difference of over £7,000 [7].

Lack of awareness of the prevalence of rare disease and limited academic training opportunities amongst clinicians are considered to be major contributors to the extended ‘diagnostic odyssey’ [8–10]. However, given the multitude of different rare diseases, providing and undertaking training in the diagnosis of each condition presents significant challenges for clinicians and researchers. Healthcare professionals need clear, evidence-based guidance on when to suspect their patient is presenting with a rare disease. This could be addressed by identifying commonalities shared by rare diseases as a group; these commonalities may be recognised as ‘red flags’ and utilised as a diagnostic tool to aid clinicians in recognising the possibility that their patient may have a rare disease.

Red flags are signs and symptoms found in the patient’s history and clinical presentation that are thought to be associated with a higher risk of serious pathology [11]. The red flag concept is typically used to identify serious pathology in patients with an otherwise common presentation, such as back pain or headache [12]. Back pain, as a recognised red flag of disease, has been utilised in The National Institute for Heath and Care Excellence’s Clinical Knowledge Summaries to aid the diagnosis of cancer, cauda equina syndrome, spinal fracture and infection [13].

The red flags concept has also been suggested to have value in other disease contexts, such as the recognition of genetic diseases in primary care settings [14]. In one study, the red flags identified included groups of congenital anomalies, early or extreme presentations of common conditions, neurodevelopmental delay or degeneration, exceptional or extreme pathology and surprising laboratory values [14]. This proposal holds significance for rare diseases, since 80% have a genetic basis [3], and implies that the red flags concept may be a relevant and useful diagnostic tool for identifying rare diseases. However, in the context of rare diseases, it is common for patients and families to carry significant psychosocial burdens in addition to physical symptoms, resulting from their unmet medical and social needs [15]. Therefore, application of the red flags concept to rare diseases requires a more holistic understanding of patients’ social, medical, psychological and physical experiences.

Given their in-depth understanding, developed through lived experience and connections with key stakeholders in the relevant disease area, rare disease patient advocacy groups are well-positioned to understand the common experiences of patients and families during the ‘diagnostic odyssey’. Therefore, this study utilises narrative-based medicine approaches by engaging rare disease patient groups to answer a questionnaire and present an account of the patient journey from symptoms to diagnosis. Through engaging the perspectives of multiple rare disease patient groups, this study identifies common experiences amongst patients with rare diseases which have the potential to aid clinicians in diagnosing rare diseases.

## Methods

### Study design

To understand the collective experiences of rare disease patients, a questionnaire was developed and shared with the wider rare disease patient group community. The questionnaire was designed by Medics for Rare Diseases (M4RD; a charity that provides education and practical tools about rare diseases as a collective group of conditions, with the aim of reducing the ‘diagnostic odyssey’ and improving the patient experience), volunteers (including doctors, rare disease patients and advocates) and eight rare disease patient advocacy groups. The questionnaire was designed to be understandable to a non-medical audience and to take less than 10 min to complete. A pilot questionnaire was completed by eight representatives from patient advocacy groups to test the design of the questionnaire, to ensure it was easy to use, and to make sure that the questions were clear and relevant.

The final questionnaire consisted of 22 questions (Table 1): 4 questions aimed to obtain information regarding the type of rare disease and patient group represented by the responder, 14 questions were designed to obtain information regarding the experiences of rare disease patients (categorised according to the question topic), 3 questions collected contact information and communication preferences, and 1 question offered responders the opportunity to provide additional information.

**Table 1:** Questionnaire summary

Question topic	Question	Type of question
Rare disease perspective	Which patient group are you representing?	Free text
	From what perspective is your interest in rare diseases?	Categorical
	What is your particular disease of interest?	Free text
	Does this disease belong to a larger group of diseases? If so, which?	Free text
Physical and psychosocial manifestations and presentation of disease	What primary medical specialty does this disease fall under?	Categorical and free text
	Which parts of the body or body systems are affected by this disease?	Categorical and free text
	Do patients with this disease have a typical physical appearance or visual clues to an underlying disease?	Categorical and free text
	Do people with this disease often have dental problems in childhood?	Categorical and free text
	Do patients with this disease lose skills or abilities they had once learnt? This is sometimes called 'regression'	Categorical
	Do patients with this disease have difficulties at school? (in the period from starting education to diagnosis)	Categorical and free text
	When does this disease present? This means when does the average patient have signs or symptoms that lead to them seeking medical help?	Categorical
	If you could choose three features of this disease that could be red flags for diagnosis, what would they be?	Free text
	Does this rare disease cause an unusual presentation of a more common disease? e.g. Type 2 diabetes in children (Alström disease) or heart attacks in young people with no risk factors (SCAD). If yes, please give an example	Categorical and free text
Patient interactions with healthcare providers	On average, how many specialists do patients with this disease see while working towards a diagnosis? Including physiotherapy, OT, dietetics, optometry, speech & language therapy and audiology	Numerical
	On average, how long does a patient with this disease have to wait for diagnosis?	Categorical
	Do patients with this disease often get misdiagnosed before receiving the correct diagnosis? Please give any common misdiagnoses given for this rare disease	Categorical and free text
Family history	Does this rare disease have a known pattern of genetic inheritance e.g. autosomal recessive/dominant or X-linked? Or do patients with this disease often have family members (close or extended) with the same disease?	Categorical and free text
	Do patients with this disease have a history or recurrent miscarriage, still birth, SIDs or unexpected complications in pregnancy in their family?	Categorical
Additional information	If you would like to elaborate on any of the above questions, please feel free to do so here	Free text
Contact information	I would like to receive information on the following: • Updates on the results on the Red Flags of Rare Disease survey • Follow-up questions about my answers to this survey • Further information about Medics4RareDiseases (you will be added to the M4RD mailing list) • None of the above	Categorical
	Please provide us with your name	Free text
	Please provide us with your email address	Free text

*Abbreviations*: *M4RD *Medics4RareDiseases, *OT* Occupational therapy, S*CAD* Spontaneous coronary artery dissection, *SIDS* Sudden infant death syndrome

The questionnaire was conducted digitally using Google Forms and was open to responses for six weeks (23rd July 2018–1st September 2018). Although the questionnaire was primarily designed to be administered online, accessibility issues were accounted for and paper versions were available on request, with the option to have a M4RD team member in attendance to ask and record the questions.

### Study participants

The questionnaire was targeted at UK-based rare disease patient groups to ensure the results were reflective of patient experiences in the UK healthcare system. Study participants were identified through snowball sampling, a purposeful method of targeting a population with characteristics that are not easily accessible [16]. Study participants were engaged through social media platforms, blogs and email newsletters of the umbrella rare disease organisations Rare Revolution Magazine, Findacure and Cambridge Rare Disease Network. Participants were predominantly representatives of rare disease patient groups, but also included healthcare professionals and family members who were identified via the sampling methodology.

Representatives from rare disease patient groups were invited to complete the questionnaire on behalf of their respective rare disease group members; the questionnaire was targeted at group representatives to understand the collective experience of a disease group rather than individuals’ stories. To collectively capture the average patient journey, only one questionnaire entry per rare disease was permitted. If a disease had distinct subtypes, respondents were asked to complete separate questionnaire entries per disease subtype.

### Ethics and consent

Data protection laws, ethical issues and consent were taken into consideration at all stages of the study. Since the questionnaire obtained data from rare disease patient groups based on ‘an average patient experience’, it did not collect individual patient information and therefore did not require ethical approval. The Medical Research Council’s decision tool was used to confirm that this project was not considered to be research, as defined by the UK Policy Framework for Health and Social Care Research [17], and this study was therefore considered to be a service evaluation*.*

Personal data was not routinely collected since the questionnaire obtained data regarding the collective experiences of patients. However, participants were invited to provide consent for sharing their contact information to allow for future communication regarding the results of the study.

### Data analysis

Data analysis was conducted on the responses that fulfilled the inclusion criteria, as described in Suppl. Table 1. For the quantitative data obtained from categorical questions, responses were analysed using IBM SPSS (version 24). Thematic analysis was conducted on the qualitative data obtained from the free-text response questions. The responses were coded in NVivo (version 12) and key themes, considered to represent a different category of related responses, were derived from the codes. Responses are presented as the proportion of total survey responses received.

## Results

### Survey respondents

A total of 116 questionnaire responses were obtained, of which 81 met the inclusion criteria and were included in the analysis. As exceptions, two responses from the same participant (representing Fibromuscular Dysplasia) were included since a second response was submitted which provided additional detail, and two responses from separate participants were included for Rett Syndrome. Overall, 79 different rare diseases were included in the statistical analysis.

Respondents were able to select multiple options when sharing which group perspective they represented. The majority of respondents (95.1%) identified as representatives of a patient group or as someone living with a rare disease. However, 76.5% identified as family members/guardians of someone with a rare disease and 8.6% identified as healthcare professionals. This indicates that, in addition to being a patient group representative or a patient with a rare disease, many respondents had professional or familial affiliations to rare diseases.

### Physical and psychosocial manifestations and presentation of disease

**Fig. 1:**
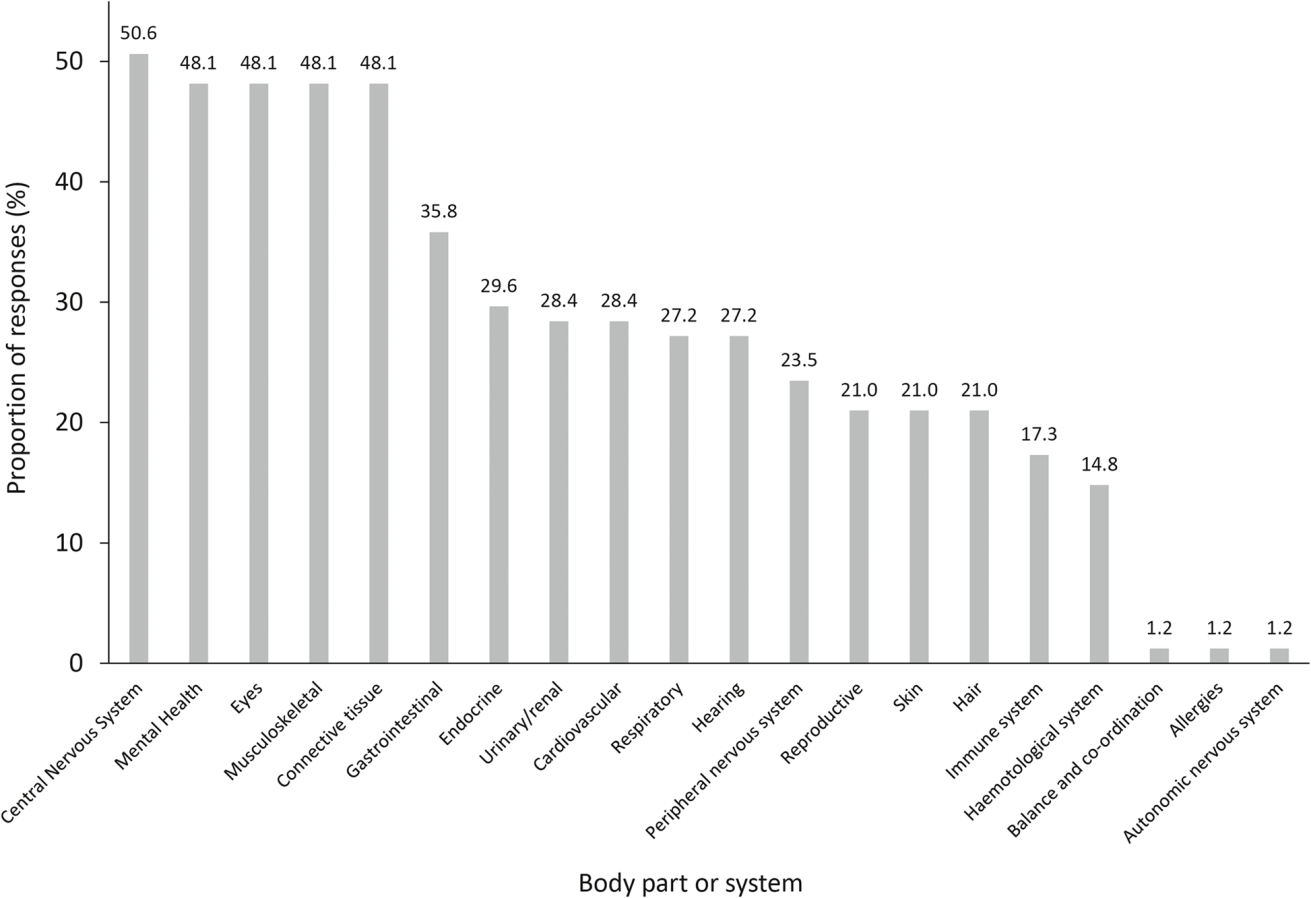
Body parts or body systems affected by rare disease. Footnotes: A total of 81 survey respondents answered this question. Respondents could provide multiple answers and percentages were calculated as a proportion of all respondents

Of the 20 different body parts or systems identified to be affected by respondents, the most common was the central nervous system, which was identified by 50.6% of respondents (Fig. 1). The next most common responses were mental health, the eyes, the musculoskeletal system and connective tissue which were all identified to be affected by 48.1% of respondents.

**Fig. 2:**
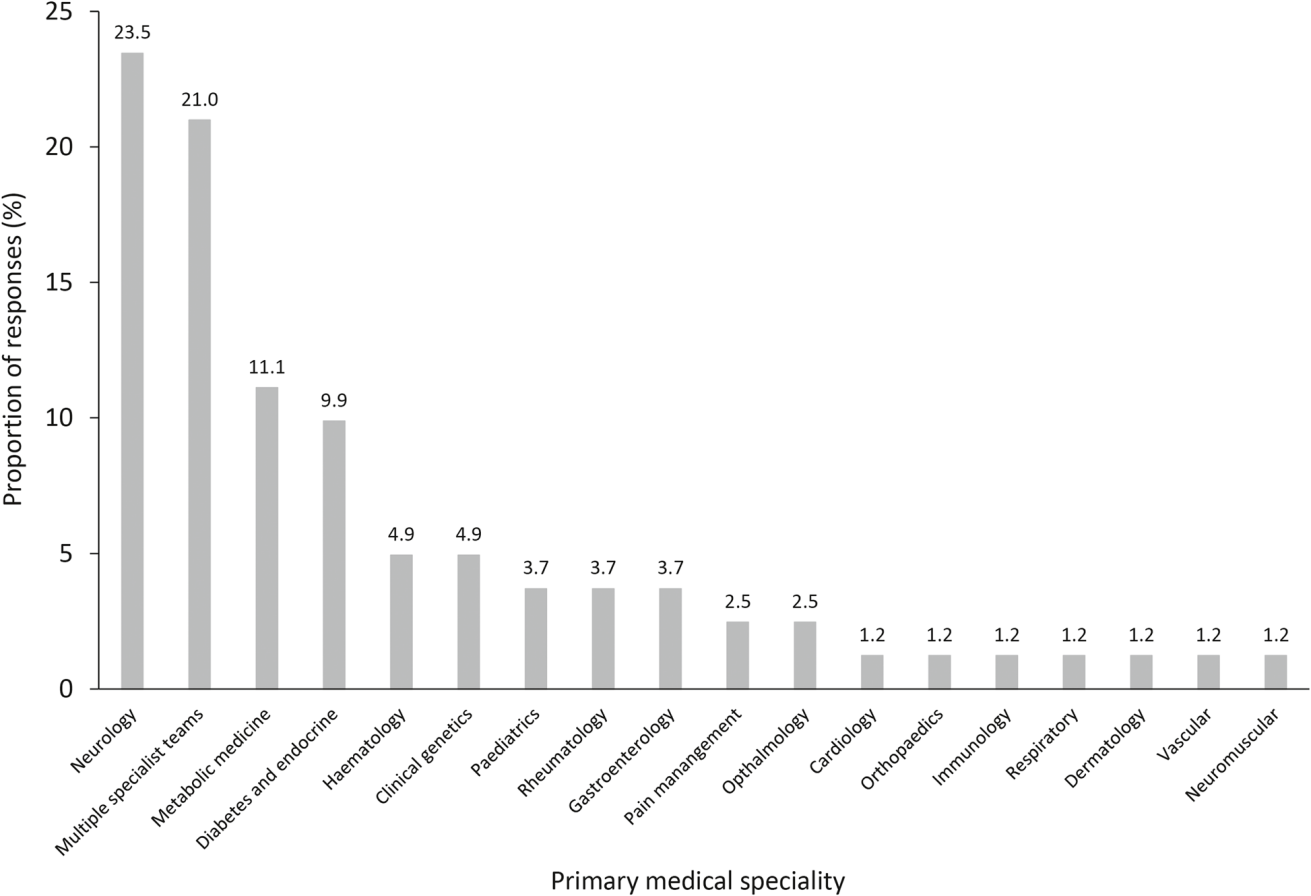
Primary medical specialty of patients with rare disease. Footnotes: A total of 81 survey respondents answered this question

In total, 18 different medical specialities were identified as the primary speciality of the given rare diseases. The most common primary medical speciality was neurology (23.5%), followed by multiple specialist teams (21.0%), metabolic medicine (11.1%) and diabetes and endocrine (9.9%) which, collectively, comprised over 65% of all responses (Fig. 2).

**Fig. 3:**
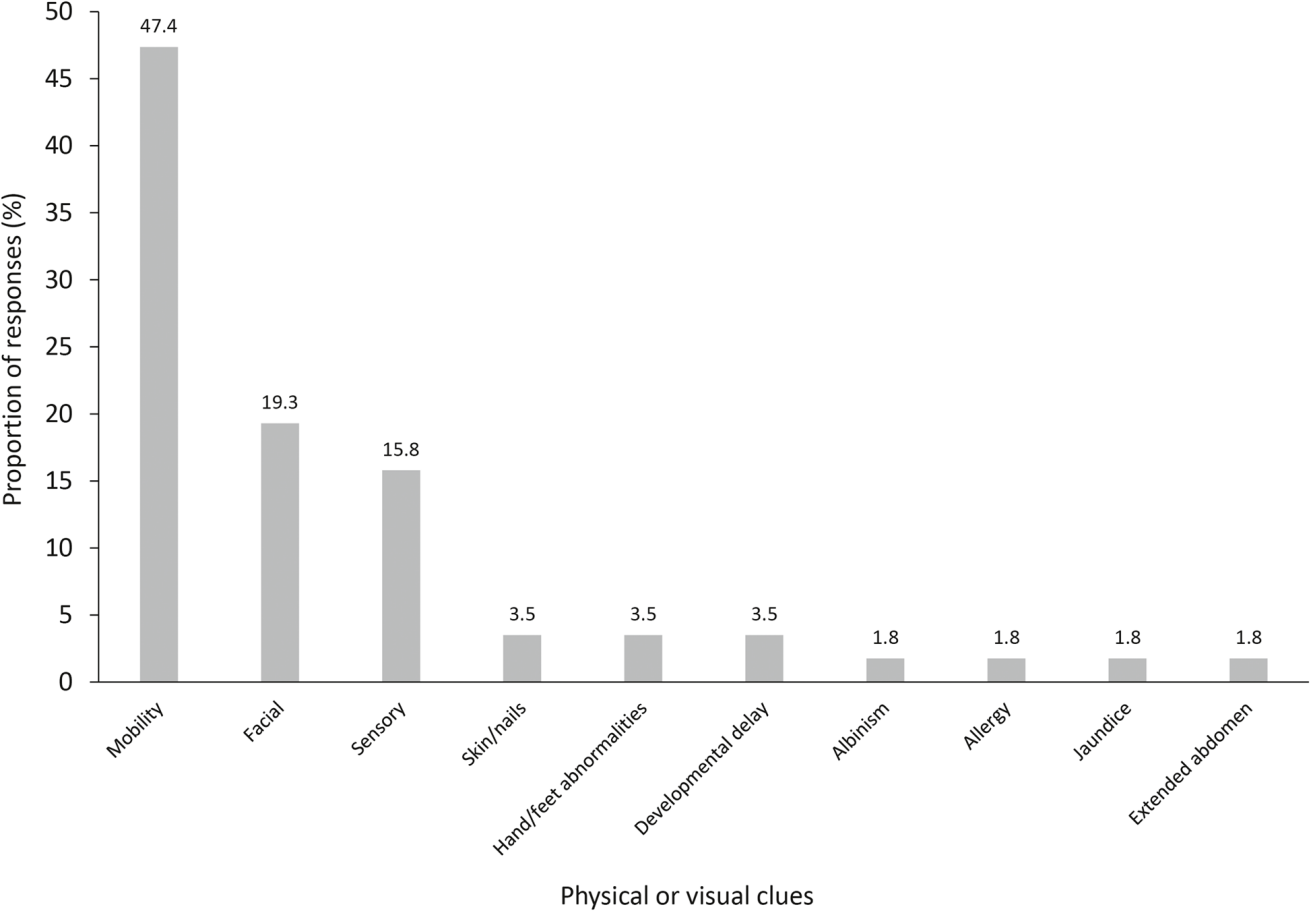
Typical physical appearances of patients with rare disease. Footnotes: A total of 57 responses were included in this analysis. Only respondents who answered ‘yes’ to the question concerning typical physical appearance or visual clues provided examples, respondents were able to provide multiple examples

The rare disease in question was considered to present with a typical physical appearance, or possess visual clues to the underlying disease, by 63.0% of respondents. Of the 57 examples of physical clues provided, the most common related to mobility (47.4%), facial (19.3%) and sensory clues (15.8%; Fig. 3). Other clues identified were less common, each contributing to ≤ 4% of all those proposed. Despite the large proportion of respondents identifying the rare disease of interest to present with physical clues, a notable proportion (37.0%) identified the rare disease to be ‘invisible’.

Dental problems in children were identified to be a typical feature of the given rare disease by 27.2% of respondents. However, nearly half (44.4%) did not identify this to be a typical feature of the rare disease and 28.4% of respondents were unable to answer this question.

Almost half (44.4%) of respondents reported the rare disease in question does not cause an unusual presentation of a more common disease, 21.0% of respondents reported it did and 14.8% reported that it sometimes did. The remaining 19.8% of respondents were unsure.

Nearly all respondents reported that their rare disease can present in childhood (95.1%), with only 4.9% reporting that it appears exclusively in adulthood. Almost half of respondents (43.2%) reported the disease appears exclusively in childhood, however, 51.9% of respondents reported the disease can present in both childhood and adulthood.

A large proportion of respondents (76.5%) identified difficulties at school to be a typical challenge for patients with the given rare disease, 17.3% did not consider this to be a challenge, 4.9% were unsure and one respondent (1.2%) identified that infants typically die before the age of two. The most common difficulties cited were physical education (21%), learning disability/difficulties (17%) and absence (15%).

In total, almost half of respondents (48.2%) reported that patients with the given rare disease lose or sometimes lose skills or abilities they had once learnt. Equally, almost half of respondents (48.2%) reported this to not be a typical experience and 3.7% of respondents were unsure.

**Fig. 4:**
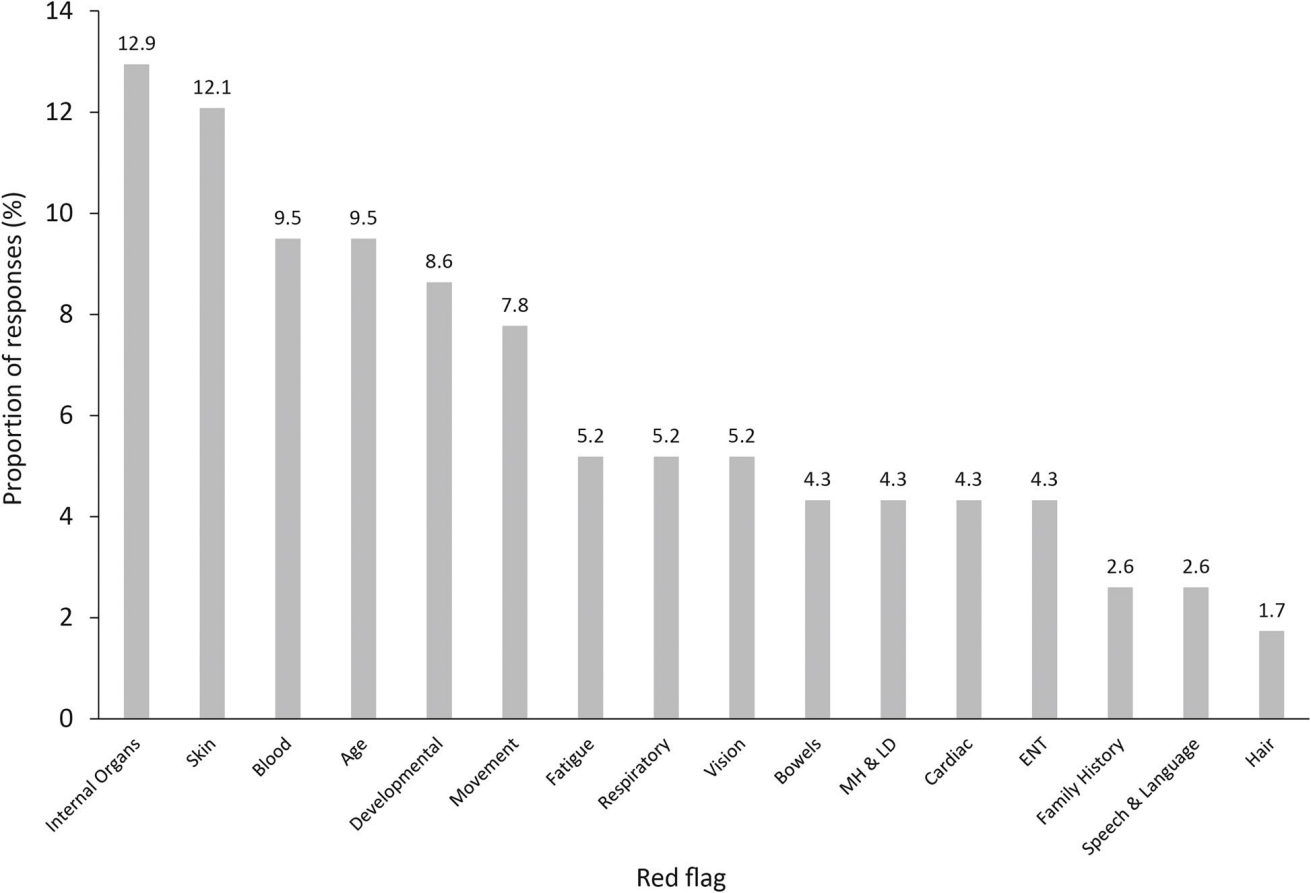
Proposed red flags of rare disease. Abbreviations: ENT: ear, nose and throat diseases; LD: learning disabilities; MH: mental health. Footnotes: A total of 116 responses from 81 respondents were included in the thematic analysis. Percentages were calculated as a proportion of all red flags identified by respondents

The thematic analysis identified 16 different categories of red flags across physical and psychosocial domains. The most common red flags proposed related to internal organs (12.9%), skin (12.1%), age (9.5%) and blood (9.5%). Other common red flags proposed included developmental (8.6%), movement (7.8%), fatigue (5.2%) and mental health and learning disabilities (4.3%; Fig. 4).

### Patient interactions with healthcare providers

When considering time to diagnosis, 16.1% of respondents reported a period of 3–6 months, 3.7% reported 6–12 months, 3.7% reported 1–2 years, 6.2% reported 2–3 years, 8.6% reported 4–5 years and 22.2% reported ≥ 5 years. The most common response was ‘unknown’ (39.5%).

A large proportion of respondents (71.6%) reported that patients typically receive a misdiagnosis before receiving the correct rare disease diagnosis. Conversely, 7.4% of respondents reported misdiagnosis to not be a typical experience and 21.0% of respondents reported it to sometimes be the case. In total, 66 different types of misdiagnoses were reported; the most common misdiagnoses included isolated mental health problems (7.7% of all misdiagnoses reported), epilepsy (5.6%), bone degeneration/arthritis/rheumatism (5.6%) and multiple sclerosis (4.9%).

**Fig. 5:**
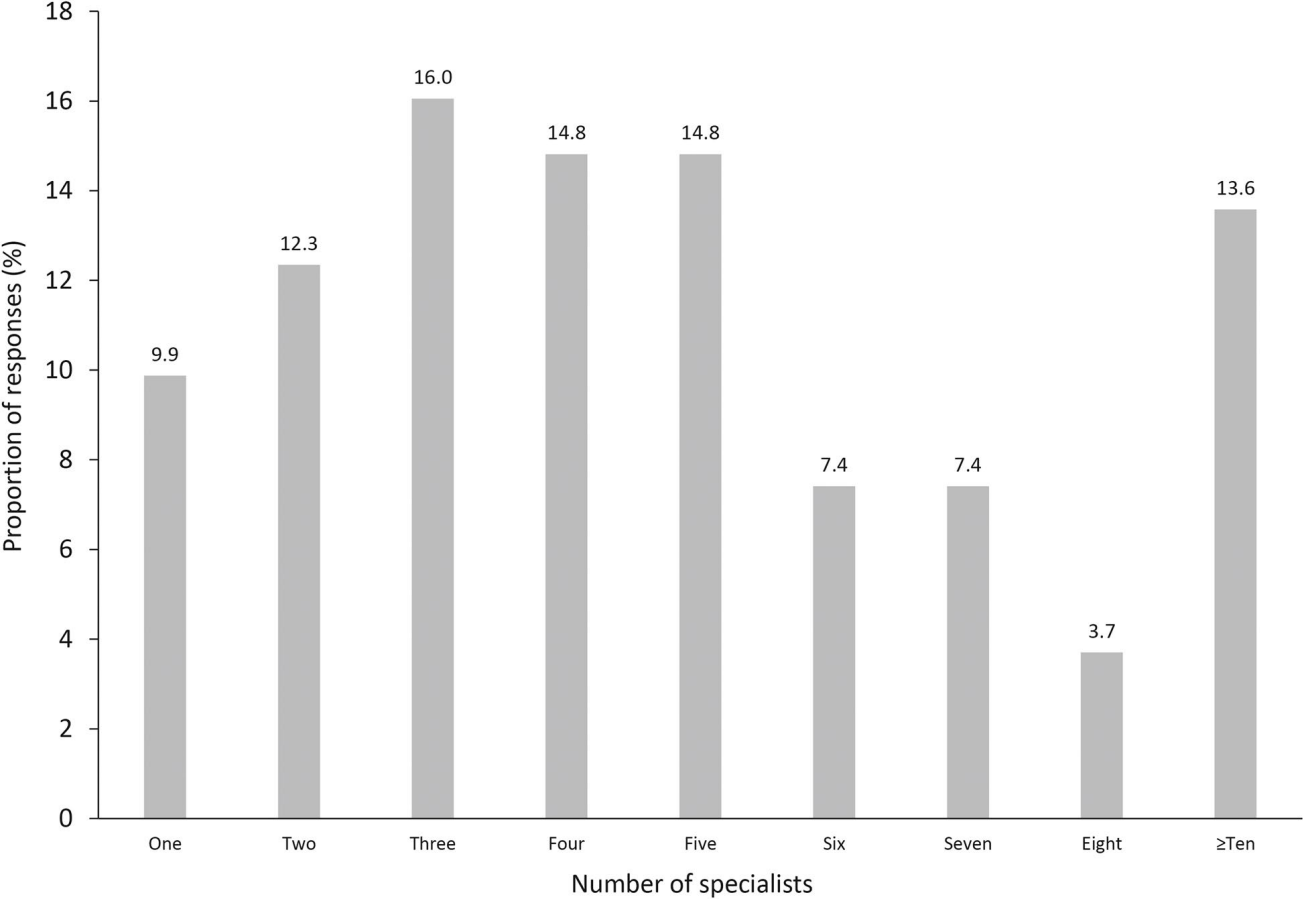
Number of specialists seen by patients with rare disease. Footnotes: A total of 81 survey respondents answered this question

The number of specialists typically seen by a patient whilst working towards a diagnosis varied greatly, from one specialist up to ≥ 10 specialists (Fig. 5). Nearly two-thirds (61.7%) of respondents reported that, on average, patients see ≥ 4 specialists whilst working towards a diagnosis, and 13.6% of patients see ≥ 10 specialists during this period. In contrast, 38.3% of patients reported seeing ≤ 3 specialists.

### Family history

A known pattern of genetic inheritance was reported in 66.7% of cases and 8.6% of respondents reported that the disease sometimes has a known pattern of genetic inheritance. Conversely, 9.9% of respondents reported it does not have a known pattern and the remaining respondents were unsure or did not respond (14.8%).

In total, 49.4% of respondents reported that there was no history of recurrent miscarriage, still birth, sudden infant death syndrome or unexpected complications in pregnancy in patients’ families. Conversely, 16.0% reported there was, or there sometimes was, and a large proportion of respondents (34.6%) reported they were unsure or it was unknown.

## Discussion

This study identified common experiences of patients with rare diseases, from symptoms through to diagnosis, and the results have been used to inform a series of red flags that can aid the recognition of when a patient may have a rare disease in non-specialist settings. As part of the questionnaire, responders were asked to propose three red flags of rare disease (Fig. 4), however, all questionnaire responses were considered collectively to determine the following seven red flags of rare diseases: multi-system involvement (3 or more); genetic inheritance pattern; continued presentation throughout childhood and adulthood; difficulties at school, especially relating to absences, difficulty participating in physical education and experiences of bullying or social isolation; multiple specialist referrals; extended period with unexplained symptoms; and misdiagnosis. These red flags include physical and psychosocial characteristics in addition to considering patients’ engagement and experiences with healthcare providers. Their diversity reflects the multiplicity of ways in which rare diseases impact patients’ lives and the many different settings in which they may present. The majority of red flags identified are of relevance to clinical and/or education environments which highlights the role of clinicians and educators in the recognition of red flags, and subsequent diagnosis of rare disease. Adopting the red flags concept in clinical practice would require clinicians to adopt a holistic view of the patient’s experiences and symptoms; the physical, psychosocial and healthcare provider interactions should be considered in combination, and the appearance of many, varied red flags may be an indicator of rare disease in itself.

For each red flag identified, recommendations for clinical practice and/or education settings are presented in Table 2. As primary care services function as patients’ first point of contact with healthcare services, they present an ideal clinical environment for the implementation of a diagnostic aid for rare disease and, crucially, they offer clinicians the opportunity to gain a holistic view of the patient’s wellbeing. It is suggested that clinicians adopt a holistic approach to assessment that considers the patient’s history, including family history, and be alert towards the common systems implicated in rare diseases. Additionally, clinicians should maintain awareness that rare diseases can present in both childhood and adulthood, and consider how patients’ interactions with healthcare systems may be indicative of rare disease, for example, if a patient has received several misdiagnoses. It is also suggested that clinicians are informed of the psychosocial red flags of rare disease in addition to physical red flags.

**Table 2:** Recommendations for red flags

Red Flag	Recommendation setting	Recommendation
Multi-system involvement (3 or more)	Healthcare settings, especially primary care	Utilise a holistic approach to assessment in clinical environments, consider the patient’s history and be aware of the most common systems affected by rare disease (CNS, Musculoskeletal, Sensory)
Genetic inheritance pattern	Healthcare settings, especially primary care	Consider the importance of family history in primary care settings and aim to understand the patient’s family history
Presentation in both childhood and adulthood	Healthcare settings, especially primary care	Maintain awareness that rare diseases can present in both childhood and adulthood and do not rule out a rare disease simply due to a patient's age
Difficulties at school e.g. especially absences, difficulty participating in physical education and bullying/social isolation	Education settings	Raise awareness amongst teachers and educators of these signs and how to recognise them. Provide advice on how educators can support parents to seek clinical assessment
Multiple specialist referrals	Healthcare settings, especially primary care and specialist care	Consider the patient’s history of engagement with healthcare providers. If a patient has seen multiple specialists and has not yet had a diagnosis, consider a rare disease
Delayed diagnosis	Healthcare settings, especially primary care	Consider the possibility of rare disease diagnosis in a patient who has not received a diagnosis after one year of seeking medical support for a health issue
Misdiagnosis	Healthcare settings, especially primary care	Consider the possibility of rare disease diagnosis in a patient who has received several misdiagnoses Do not rule out a rare disease simply because a patient has an established diagnosis of epilepsy or arthritis; given that these may be secondary processes

*Abbreviation*: *CNS* Central nervous system

Education settings are proposed to have a role in identifying individuals presenting with rare disease therefore it is suggested that awareness of rare diseases should be raised amongst teachers and other educators. For example, the physical and psychosocial red flags of rare disease, behavioural difficulties indicating rare disease and how to support parents to seek clinical assessment should be explored. This is further supported by a previous survey of educators in Spain, where approximately 28% and 48% answered “Not at all” for the questions “I know the warning signs a student with a possible rare disease may present” and “I know how to clinically diagnose a rare disease”, respectively [18].

Given that 71.6% of respondents identified their respective rare disease to affect three or more body parts or systems, it is suggested that multi-system involvement should be considered as a red flag of rare disease. In addition, the large number of different medical specialities proposed to be primary medical specialities (*n* = 18), and the inclusion of allied healthcare professions within this, highlights the importance of thorough history taking and the need to consider the wide range of potential symptoms and medical specialities that may be relevant. Additionally, it is notable that the second most common ‘discipline’ identified was multiple specialist teams, which indicates that rare diseases typically cause varied physical and psychosocial impacts. Over a third of respondents reported their rare disease to be invisible, which further emphasises the variability in rare disease presentation and is important to consider since ‘invisible’ diseases are typically more difficult to identify, reinforcing the importance of thorough history taking and understanding the patient’s experience. Despite the importance of taking a thorough patient history described here, it should be acknowledged that this is reliant on patients disclosing all relevant information to their healthcare provider, which may in itself become a barrier to diagnosis. This underlines the importance of effective interactions between patients and healthcare professionals, including education of the patient, psychological support and trust-building, to aid in the diagnosis of rare diseases [19].

The importance of considering patient history is also emphasised by the results for questions concerning disease presentation and problems in childhood. Over 75% of respondents reported that patients experienced difficulties at school, the most common issues being difficulty participating in physical education, learning disabilities and regular/prolonged absences from school; observation of these factors could encourage clinicians to consider the possibility of a rare disease in a young patient. Furthermore, although current literature reports that approximately 70% of rare diseases present exclusively in childhood, in this analysis, over half of respondents reported that disease can present in both childhood and adulthood [2]. Therefore, clinicians should maintain awareness that rare diseases can present in adulthood or may remain undiagnosed until adulthood. However, given that the results for this question vary from the literature, further study should be conducted to understand whether specific rare diseases present exclusively in childhood and how awareness of this could be used to aid diagnosis in clinical settings.

Respondents also reported common experiences when interacting with healthcare providers; over 75% of respondents reported that patients experience three or more referrals, which implies patients are frequently referred to specialists who were unable to determine an appropriate diagnosis. Furthermore, whilst the varied results indicate it is difficult to determine an average time to diagnosis, patients typically experience a delayed diagnosis, with over 20% of respondents reporting a time to diagnosis of over five years. Given the well-established ‘diagnostic odyssey’ associated with rare diseases [20], this is an unsurprising but nevertheless critical result which demonstrates the immense challenge of timely diagnosis. Delayed diagnosis can also be compounded by misdiagnoses. In this study, over 70% of respondents reported it was typical for patients to be labelled with an alternative diagnosis which poses a risk to patient safety given that misdiagnoses can lead to the premature ending of the diagnostic work-up, preventing further assessment or evaluation for alternative aetiology. These findings are in agreement with a report for the Department of Health and Social Care which postulates that, on average, a patient may see five doctors and have three misdiagnoses prior to formal diagnosis [20]. However, it is important to consider alternative explanations for the large proportion of patients reporting misdiagnoses; it may be that respondents have misinterpreted clinical investigations conducted to rule out common causes of their symptoms to be misdiagnoses and further work is needed to understand patient group perceptions of misdiagnosis.

Reported misdiagnoses varied widely and included both physical and mental health conditions across all specialities and body systems. Epilepsy and arthritis were amongst the most common misdiagnoses reported; however, as evidenced by the disease processes of adrenoleukodystrophy and alkaptonuria, these conditions can be secondary processes, or part of a syndrome, and do not necessarily constitute the final diagnosis [21, 22]. This highlights how secondary disease processes can be incorrectly diagnosed as primary conditions and have the potential to exacerbate the rates of misdiagnosis amongst patients with rare disease. Additionally, it is notable that the most common misdiagnosis reported was isolated mental health problems (7.7% of all misdiagnosis reported) which may lead to premature ending to the diagnostic work-up, allowing disease progression and potential iatrogenic development of mental health disorders as a result of their physical health burden. However, as described in previous reports published by Rare Disease UK and Genetic Alliance UK, receiving a mental health diagnosis in the context of an undiagnosed rare disease may be distressing to a patient since they may be given the impression that their clinician perceives their symptoms as fictitious [23, 24]; it is therefore important that clinicians have an awareness of the psychosocial burden of rare diseases. Whilst misdiagnosis is a challenging and complex issue to overcome in clinical practice, there is a role for increasing awareness of the rates of misdiagnosis and common misdiagnoses amongst clinicians.

Given that this study was designed as a service evaluation, the red flags identified do not present generalisable findings but rather present information that can be used to inform decision-making in clinical settings. The authors acknowledge that the efficacy of implementing these actions has not been tested and suggest that the findings of this study are used to inform a diagnostic framework or tool, designed to optimise early recognition of rare disease, that can be implemented and tested in primary care settings.

Strengths of this study include the range of rare disease groups sampled; the study methodology was designed to obtain a broad range of rare diseases, so the results were reflective of the collective experiences of rare disease patients, as opposed to one group of rare disease patients. In total, 79 different rare diseases were captured by the survey and whilst this only represents a small proportion of all rare diseases, it was sufficiently broad to capture common experiences across different rare disease groups. Furthermore, the restriction to only include rare disease groups based in the UK ensured the results were representative of the experiences of rare disease patients in the UK and therefore, applicable to the UK healthcare setting. The use of qualitative analysis enabled deeper insights into the respondents’ narrative accounts to be explored, which was a key objective of the study.

Limitations of the study include the sampling methodology employed which was non-random and therefore subject to bias. The sample also included a mix of patient representatives and healthcare professionals, which may contribute to some level of bias in the study findings. However, it should be noted that this approach was taken in order to gain a holistic view of patient experiences with rare disease diagnosis. Furthermore, although the questionnaire clearly described its objective to identify the common experiences of rare disease patients, respondents may be inclined to answer questions based on their own personal experiences, rather than shared, collective experiences, which would limit the utility of the results for informing a diagnostic tool. Regarding the analysis, there is a level of subjectivity involved in thematic analysis and therefore there was potential for the analysts’ personal experiences/bias to influence the results of the analysis. Lastly, we acknowledge the time interval between collection of the data in 2018 and publication of these results and encourage healthcare professionals to take these findings together with their lived experiences and in the context of their current practice.

Given the role of the rare disease community in generating these results and the importance of transparent information sharing, especially regarding the ‘diagnostic odyssey’, it is important that these results are disseminated to the rare disease community and wider clinical audiences. Given that the utility of the red flags proposed in this publication has not been tested in clinical practice, further research should be conducted to understand the efficacy of the red flags concept in diagnosing rare diseases in primary care settings.

## Conclusions

Whilst rare diseases are a heterogenous group of conditions, identification of key commonalities across physical and psychosocial domains, in addition to understanding patients’ history and interactions/experiences with healthcare providers may support non-specialist practitioners to suspect rare disease diagnoses, particularly in primary care. The red flags proposed here could be used to inform a clinical decision-making tool to optimise early recognition of rare disease in primary care settings, presenting the opportunity to minimise the challenges of the ‘diagnostic odyssey’ and improve the patient experience. However, further work is needed to understand the optimal design and implementation of such a tool.

### Supplementary Information

**Supplementary Material 1.**

## Data Availability

The datasets generated and/or analysed during the current study are available from the corresponding author on reasonable request.
