# Supplementary material for: A quantitative and qualitative analysis of patient group narratives suggests common biopsychosocial red flags of undiagnosed rare disease
Source: Orphanet J Rare Dis. 2024 Apr 19;19:172. doi: 10.1186/s13023-024-03143-8 (PMC11031885; doi:10.1186/s13023-024-03143-8)
Supplement: Supplementary file 1 — Supplementary Material 1. [file 13023_2024_3143_MOESM1_ESM.docx]

SUPPLEMENTARY APPENDIX

Suppl. Table 1: Inclusion and exclusion criteria

| Inclusion criteria | Exclusion criteria |  |
| --- | --- | --- |
| - Respondents submitted on behalf of patient groups - Respondents from rare disease groups based in the UK | - Individual respondents who were not affiliated to patient groups - Duplicate responses representing the same disease^a^ - Responses withdrawn by the responder - Respondents from rare disease groups located outside of the UK | |

**Footnotes:** ^a^Where multiple responses were submitted for one disease, the response from the email address affiliated to a patient group was selected.

Suppl. Table 2: List of abbreviations

| **Abbreviation** | **Definition** |  |
| --- | --- | --- |
| CNS | Central nervous system | |
| LD | Learning disabilities | |
| M4RD | Medics4RareDiseases | |
| MH | Mental health | |
| OT | Occupational therapy | |
| SCAD | Spontaneous coronary artery dissection | |
| SIDS | Sudden infant death syndrome | |
